# Heated Tobacco Products Have Reached Younger or More Affluent People in Japan

**DOI:** 10.2188/jea.JE20190260

**Published:** 2021-03-05

**Authors:** Ayaka Igarashi, Jun Aida, Taro Kusama, Takahiro Tabuchi, Toru Tsuboya, Kemmyo Sugiyama, Takafumi Yamamoto, Ken Osaka

**Affiliations:** 1 Department of International and Community Oral Health, Tohoku University Graduate School of Dentistry, Sendai, Japan; 2 Cancer Control Center, Osaka International Cancer Institute, Osaka, Japan

**Keywords:** heated tobacco products, electronic nicotine delivery devices, smoking, characteristic, socioeconomic status

## Abstract

**Background:**

The trend of the diffusion of heated tobacco products (HTPs) is a great concern because HTPs have become available worldwide. This study examined the sociodemographic characteristics of HTPs users in Japan, where HTPs were first launched.

**Methods:**

This cross-sectional study used data from an online survey conducted in 2017. A total of 4,926 participants, aged 20–69 years, were included. The dependent variable was the type of tobacco products used. The independent variables were age and equivalent income. Two analyses estimated the odds ratios (ORs) for 1) being smokers compared to “non-smokers,” and 2) being “HTP smokers” compared to “only combustible cigarette smokers.” Analyses were stratified by sex. Educational attainment and occupation were also used in the sensitivity analyses.

**Results:**

The percentages of “non-smokers,” “only combustible cigarette smokers,” and “HTP smokers” were 82.8%, 14.2%, and 3.0%, respectively. When compared to the oldest participants (aged 60–69), the youngest participants (aged 20–29) tended to be “HTP smokers” (OR 7.90; 95% confidence interval [CI], 3.09–20.22 for men and OR 9.28; 95% CI, 2.14–40.28 for women). Compared to participants with the lowest incomes (<2 million), those with the highest incomes (≥4 million) tended to use HTPs (OR 2.93; 95% CI, 1.56–5.49 in men and OR 1.82; 95% CI, 0.73–4.54 in women). These trends were consistent when analyses included only smokers. There were consistent results in other SES measurements, including educational attainment and occupation.

**Conclusions:**

Younger or more affluent people tended to use HTPs, although smoking rates among these populations were generally lower. New tobacco control efforts are required.

## INTRODUCTION

Tobacco smoking is the most important attributable risk factor for mortality in many diseases, such as cancer and cardiovascular disease.^1^ It is estimated that the percentage of global deaths attributable to smoking was 11.5% in 2015: 6.4 million people worldwide.^2^ Despite the decline in overall smoking rates in some countries, the disease burdens related to smoking are still tremendous.^3^ For example, death due to smoking remains the main cause of mortality in Japan, although smoking rates have declined.^2^^,^^4^^,^^5^ The reduction in the smoking rate is still an important public health policy globally because smoking is a major and preventable risk factor for individuals and for society.

Recently, new tobacco products are being diffused worldwide, and heated tobacco products (HTPs) have become widely available.^6^ On April 30, 2019, the United States Food and Drug Administration (FDA) allowed the sale of IQOS, which is a type of HTP, in the American market.^7^ HTPs were first launched in Japan and Italy as test markets, and the trend of the diffusion of HTPs in these countries is of great concern.^8^ HTPs are a type of electronic tobacco product that produces aerosols containing nicotine and other chemicals by vaporizing specific tobacco leaves.^9^ In Japan, HTPs are defined as tobacco products and are regulated by tobacco business law because the HTPs use tobacco leaves. By contrast, e-cigarettes are not popular in Japan because e-cigarettes containing nicotine have been banned for sale. After the sale of HTPs in Japan, they have been rapidly diffusing.^8^ Currently, three types of HTPs—IQOS, Ploom Tech, and glo—are sold, and the prevalence of use of these tobacco products has increased from 1.4% in 2015 to 4.7% in 2017.^8^

**Figure 1.**
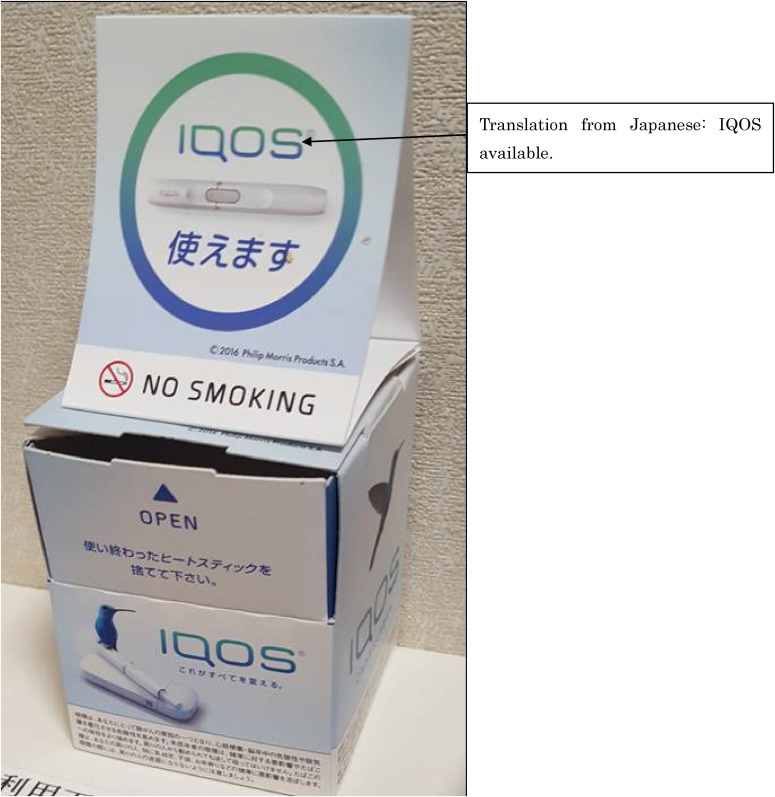
An example of a new image strategy for heated tobacco products (HTPs): A dust box used for HTP sticks in a hotel room prohibiting traditional combustible cigarette use. It displays “No smoking” and “IQOS is available” (photo by JA at a hotel in 2019).

The spread of HTPs and the characteristics of their users must be studied to monitor tobacco control efforts.^10^ Despite the lack of epidemiological evidence for the reduction of the harmful effects of HTPs, they have been sold at relatively higher prices with smoke-free and low-risk images.^11^^–^^13^ Even the term “No smoking” is used in their images (Figure 1). These characteristics possibly appeal to different populations of traditional combustible cigarette users, and if so, new tobacco control efforts are required. Previous substantial studies have reported socioeconomic inequalities in traditional combustible cigarette use^14^^–^^17^; socioeconomically disadvantaged populations tend to have higher smoking rates. Therefore, the policy of raising tobacco taxes, which is the most effective tobacco control policy,^18^ has the greatest health benefit for socioeconomically disadvantaged populations.^19^ However, in relation to HTPs, only a few studies have examined the difference of use based on socioeconomic status (SES). Previous studies examining SES and HTPs use have reported a non-significant association with SES, although people with a higher SES more frequently used HTPs.^6^^,^^20^ However, there is a possibility that SES differences related to HTPs use differ compared to previous research because the percentage of people using HTPs has increased with time and due to the advertisement efforts of HTP companies. The aim of the present study is to determine the association between sociodemographic characteristics of HTP users in Japan.

## METHODS

### Settings and participants

This was a cross-sectional study that used data from an online survey from January 28, 2017, to February 1, 2017, in Japan. The participants in this online survey were randomly selected from a registry established by an Internet research company. The total number of participants was 5,000. The same number of participants in each sex and age category on a scale of 10 years from 20–69 were included in the survey from all 47 prefectures in Japan. All participants answered all the content of the questionnaires after reading the explanation about participation in the survey and agreed to participate. The questionnaire asked about smoking status and type of tobacco products used, such as traditional combustible cigarettes, HTPs, e-cigarettes, or others. In addition, some demographic and SES questions were also asked. The dependent variable was the type of tobacco products used, and it was categorized as “non-smokers,” “smokers using only traditional combustible cigarettes,” and “smokers using HTPs, including using together with other tobacco products.” Therefore, fewer respondents, who “use only other tobacco products” and “use combustible cigarettes and other tobacco products,” were excluded from the analysis (*n* = 74) in order to compare to smokers who only use combustible cigarettes and smokers who use HTPs. Finally, 4,926 participants were included in the present analysis.

This study protocol was approved by the Research Ethics Committee of Tohoku University. (ID: 2016-3-028)

### Dependent variables

We used smoking status, including the type of tobacco products used, as a dependent variable. Smoking status was based on the use of tobacco products during the previous 30 days. Respondents were asked to select tobacco products they use from the following choices: combustible cigarettes; HTPs; and other tobacco and tobacco-like products, including e-cigarettes, cigars, chewing tobacco, and water pipes. In the questionnaire, we provided each HTP name (IQOS, Ploom Tech, and glo), and respondents who selected at least one of those three products were categorized as HTP users. Smoking status was categorized into three groups: non-smokers, smokers who only use combustible cigarettes (only combustible cigarette smokers), and smokers who only use HTPs or HTPs and other tobacco products (HTP smokers).

### Independent variables

The independent variables were age and SES. We used equivalent income as the main indicator of SES. The reason is that HTPs have been sold at relatively higher prices, and the price is most relevant to the income. However, we also used educational attainment and occupation as SES indices as the sensitivity analysis, and their results were described in the supplemental file. We categorized age into five groups as follows: 20–29 years old, 30–39 years old, 40–49 years old, 50–59 years old, and 60–69 years old. Equivalent income was categorized into five groups: <2 million Japanese yen (JPY); 2.00–2.99 million JPY; 3.00–3.99 million JPY; and ≥4 million JPY. One hundred JPY is almost equal to 1 United States dollar.

### Statistical analysis

We conducted two types of analysis: 1) estimating odds ratios (ORs) for smokers compared to “non-smokers”; and 2) estimating ORs for “HTP smokers” compared to “only combustible cigarette smokers.” For the first analysis, multinomial logistic regression analysis was used to calculate the adjusted ORs with 95% confidence intervals (CIs) of age and equivalent income for “only combustible cigarette smokers” and “HTP smokers” compared to “non-smokers.” For the second analysis, logistic regression analysis was used to calculate the adjusted ORs and 95% CIs of age and equivalent income for “HTP smokers” compared to “only combustible cigarette smokers.” These analyses were stratified by sex. As the sensitivity analysis, instead of equivalent income, educational attainment or occupation was included in the models. All analyses used STATA MP version 15.0 (Stata Corp, College Station, TX, USA).

## RESULTS

Table 1 shows the basic characteristics of the study participants according to their smoking status, stratified by sex. Among the 4,926 participants, the number of “non-smokers” was 4,077 (82.8%). There were 700 (14.2%) “only combustible cigarette smokers” and 149 (3.0%) “HTP smokers.” Men, younger participants, and participants with higher incomes tended to be HTP smokers. The distribution of educational attainment and occupation according to smoking status are described in eTable 1.

**Table 1.** Basic characteristics of participants according to smoking status and type of tobacco product

		Total (*n* = 4,926)		Non-smokers (*n* = 4,077)		Only combustible cigarette smokers (*n* = 700)		HTP smokers^a^ (*n* = 149)	
		*n*	%	*n*	%	*n*	%	*n*	%
Men	Total	2,443	100	1,858	76.1	472	19.3	113	4.6
	Age, years								
	20–29	486	19.9	390	21.0	54	11.4	42	37.2
	30–39	489	20.0	375	20.2	84	17.8	30	26.5
	40–49	492	20.1	355	19.1	115	24.4	22	19.5
	50–59	486	19.9	358	19.3	114	24.2	14	12.4
	60–69	490	20.1	380	20.4	105	22.2	5	4.4

	Equivalent income, JPY/year								
	<2 million	571	23.4	463	24.9	95	20.1	13	11.5
	2.00–2.99 million	533	21.8	407	21.9	105	22.3	21	18.6
	3.00–3.99 million	502	20.5	363	19.5	111	23.5	28	24.8
	≥4 million	837	34.3	625	33.7	161	34.1	51	45.1

Women	Total	2,483	100	2,219	89.4	228	9.2	36	1.4
	Age, years								
	20–29	496	20.0	454	20.5	24	10.5	18	50.0
	30–39	495	19.9	453	20.4	36	15.8	6	16.7
	40–49	497	20.0	427	19.2	63	27.6	7	19.4
	50–59	497	20.0	435	19.6	59	25.9	3	8.3
	60–69	498	20.1	450	20.3	46	20.2	2	5.6

	Equivalent income, JPY/year								
	<2 million	673	27.1	593	26.7	72	31.6	8	22.2
	2.00–2.99 million	576	23.2	528	23.8	43	18.9	5	13.9
	3.00–3.99 million	549	22.1	491	22.1	47	20.6	11	30.6
	≥4 million	685	27.6	607	27.4	66	28.9	12	33.3

HTP, heated tobacco product.

^a^HTP smokers include smokers using only HTPs or HTPs and other tobacco products.

Table 2 shows the ORs of age and equivalent income for “only combustible cigarette smokers” and “HTP smokers” compared to “non-smokers” using multinomial logistic regression analysis. Among men, younger and more affluent participants used HTPs more significantly. However, “only combustible cigarette smokers” were significantly older in age, and an income difference was not observed. When comparing the oldest participants (aged 60–69) to the youngest participants (aged 20–29), the ORs for “only combustible cigarette smokers” and “HTP smokers” were in opposite directions, 0.50 (95% CI, 0.35–0.71) and 7.90 (95% CI, 3.09–20.22), respectively. Compared to participants with the lowest incomes (<2 million), those with the highest incomes (≥4 million) were 2.93 (95% CI, 1.56–5.49) times more likely to use HTPs, although social differences were not clear for only combustible cigarette smoking.

**Table 2.** Odds ratios of age and equivalent income for smokers (only combustible cigarette or HTP) versus non-smoker using multinomial logistic regression analysis

		OR for being only combustible cigarette smokers	OR for being HTP smokers^b^
		aOR^a^ (95% CI)	aOR^a^ (95% CI)
Men	Age, years		
(*n* = 2,443)	20–29	0.50 (0.35–0.71)	7.90 (3.09–20.22)
	30–39	0.79 (0.57–1.09)	5.48 (2.10–14.32)
	40–49	1.15 (0.85–1.56)	4.18 (1.56–11.19)
	50–59	1.14 (0.84–1.55)	2.51 (0.89–7.09)
	60–69	1.00 (reference)	1.00 (reference)

	Equivalent income, JPY/year		
	<2 million	1.00 (reference)	1.00 (reference)
	2.00–2.99 million	1.27 (0.93–1.73)	1.74 (0.86–3.53)
	3.00–3.99 million	1.46 (1.07–1.99)	2.67 (1.36–5.25)
	≥4 million	1.19 (0.89–1.58)	2.93 (1.56–5.49)

Women	Age, years		
(*n* = 2,483)	20–29	0.51 (0.31–0.85)	9.28 (2.14–40.28)
	30–39	0.78 (0.49–1.23)	2.94 (0.59–14.65)
	40–49	1.46 (0.98–2.19)	3.54 (0.73–17.17)
	50–59	1.33 (0.88–2.00)	1.44 (0.24–8.66)
	60–69	1.00 (reference)	1.00 (reference)

	Equivalent income, JPY/year		
	<2 million	1.00 (reference)	1.00 (reference)
	2.00–2.99 million	0.65 (0.44–0.97)	0.75 (0.24–2.30)
	3.00–3.99 million	0.74 (0.50–1.09)	1.84 (0.73–4.64)
	≥4 million	0.81 (0.57–1.16)	1.82 (0.73–4.54)

aOR, adjusted odds ratio; CI, confidence interval; HTP, heated tobacco product.

^a^Adjusted for age and equivalent income.

^b^HTP smokers: smokers using only HTPs or HTPs and other tobacco products.

Table 3 shows the ORs for “HTP smokers” compared to “only combustible cigarette smokers” using logistic regression analysis. For both men and women, the youngest participants were significantly being “HTP smokers,” with ORs of 16.38 (95% CI, 6.07–44.17) and 22.26 (95% CI, 4.58–108.13), respectively. Affluent participants tended to be “HTP smokers.” Among men, compared to participants with the lowest incomes (<2 million), those with the highest incomes (≥4 million) were 2.48 (95% CI, 1.23–5.01) times more likely to use HTPs. Among women, the OR of the participants with 3.00–3.99 million was 3.34 (95% CI, 1.11–10.04) compared to participants with the lowest incomes.

**Table 3.** Odds ratios of age and equivalent income for HTP smokers versus only combustible cigarette smokers using logistic regression analysis

		OR for being HTP smokers^b^
		aOR^a^ (95% CI)
Men	Age, years	
(*n* = 585)	20–29	16.38 (6.07–44.17)
	30–39	6.93 (2.57–18.72)
	40–49	3.68 (1.34–10.12)
	50–59	2.25 (0.78–6.52)
	60–69	1.00 (reference)

	Equivalent income, JPY/year	
	<2 million	1.00 (reference)
	2.00–2.99 million	1.41 (0.64–3.11)
	3.00–3.99 million	2.20 (1.03–4.71)
	≥4 million	2.48 (1.23–5.01)

Women	Age, years	
(*n* = 264)	20–29	22.26 (4.58–108.13)
	30–39	4.13 (0.77–22.11)
	40–49	2.90 (0.56–14.94)
	50–59	1.19 (0.19–7.51)
	60–69	1.00 (reference)

	Equivalent income, JPY/year	
	<2 million	1.00 (reference)
	2.00–2.99 million	1.23 (0.34–4.41)
	3.00–3.99 million	3.34 (1.11–10.04)
	≥4 million	2.54 (0.88–7.35)

aOR, adjusted odds ratio; CI, confidence interval; HTP, heated tobacco product.

^a^Adjusted for age and equivalent income.

^b^HTP smokers: smokers using only HTPs or HTPs and other tobacco products.

Analyses using educational attainment and occupation as SES measurements are shown in the supplemental material. The results of these sensitivity analyses were consistent with the results of equivalent income. Those with “Part-timer” and “Inoccupation” had significantly less HTPs users with respective ORs of 0.18 (95% CI, 0.04–0.77) and 0.27 (95% CI, 0.10–0.74) than those with “Clerical” among men (eTable 2). Those with “University and Graduate school” were significantly more likely to use HTPs (OR 4.12; 95% CI, 1.55–11.00) than those with “Junior high school and High school” among women (eTable 3).

## DISCUSSION

To the best of our knowledge, this study is the first to examine the sociodemographic differences in HTP users several years after the launch of HTPs. Younger people and more affluent people frequently use HTPs. Although smoking rates are lower among younger people and people with higher SESs,^16^ HTPs appear to disrupt the reduction in the smoking rate in Japan. These results suggest the direction of tobacco companies’ marketing efforts. Public health sectors must respond to these efforts to reduce smoking rates, including HTPs. There were consistent results in three SES measurements (income, education, and occupation), though income showed the clearest results.

The present results of the positive association between SES and HTPs use were not consistent with a previous study. Only one study has examined the association between SES and HTPs use, and it found a non-significant association of educational attainment as a proxy for SES on HTPs use.^21^ The cause of this result was that HTPs launched in 2014, and their study was conducted in 2015. In addition, a smaller sample size of HTP users resulted in lower statistical power.^21^ In the study, there were only 40 HTP users out of 7,338 participants.^21^ Some studies have claimed that a positive association exists between the use of e-cigarettes, one of the new cigarettes, and SES,^22^^,^^23^ although few studies have been conducted that focus on HTPs. However, e-cigarettes with nicotine have been legally prohibited in Japan, and the number of e-cigarette users is small (*n* = 86) in the present study. Therefore, we did not observe e-cigarettes users in this study. Additional studies are needed to determine the characteristics of HTP users when the prevalence of HTP users will have increased.

The present result indicating that younger or more affluent people use HTPs. Users of HTPs are different from those of traditional combustible cigarettes users, which may be explained by the advertisement and marketing strategy of HTPs by tobacco companies. Historically, to promote smoking among younger people, tobacco companies have appealed to a healthier image.^24^ In spite of the more addictive effect of menthol tobacco due to interaction with nicotine, tobacco companies misled younger people that menthol tobacco was healthier.^24^ Similarly, in relation to HTPs, tobacco companies claim that their health risks are smaller compared to traditional combustible cigarettes.^25^ Philip Morris International, which produces IQOS, has claimed about reduced harm as follows: ‘switching completely from conventional cigarettes to the IQOS system’ (1) ‘can reduce the risks of tobacco-related diseases;’ (2) ‘significantly reduce[s] your body’s exposure to harmful or potentially harmful chemicals;’ and (3) ‘presents less risk of harm than continuing to smoke cigarettes.’^26^ This healthier image is also considered to be effective for people with higher SES. Previous studies have reported that those with higher SES preferred lower health risk behaviors.^27^^,^^28^ Therefore, among smokers, more affluent people might choose HTPs because these products claim that their health risks are smaller than combustible cigarettes, despite the companies only emphasizing specific harmful substances without any consideration of multiple exposures to toxic substances in HTPs. To reduce the harmful image and to appeal to younger people, packaging and marketing of IQOS imitate non-tobacco products.^29^ IQOS were sold in smartphones-like packages, and IQOS flagship stores are similar to high-end technology brand stores, such as Apple or Microsoft stores.^29^ Another possible reason for the present study results is that poor people cannot migrate from combustible cigarettes to HTPs due to the higher prices of HTPs compared to combustible cigarettes.^11^^,^^12^

The present results suggest several implications. HTPs appear to disrupt the reduction in the smoking rate, especially among younger people and people with higher SESs in Japan. As mentioned above, this situation is considered to be caused by advertisements by tobacco companies, which overemphasize the safety of HTPs, despite the lack of epidemiological evidence. Younger people and more affluent people tended to use HTPs, although smoking rates among these populations were generally lower. Recently emerging HTPs seem to disrupt the reduction in the smoking rate among these populations. New tobacco control efforts tackling HTPs are required. Recent studies have indicated the harmful effects of HTPs.^30^ Compared to combustible cigarettes, in HTPs, levels of some harmful substances were lower but several substances were higher.^31^ A study reported that the nicotine concentration in the blood after smoking HTPs was similar to that of combustible cigarettes.^32^ A systematic review concluded that HTPs exposed users and bystanders to toxic substances.^33^^–^^35^ These studies suggest the possible harmful effects of the passive smoking of HTPs. The WHO report published in July 2019, claimed that heated tobacco contains the same harmful substances as combustible cigarette and does not necessarily reduce health risks.^36^ In addition, it pointed out that the harm of passive smoking cannot be denied.^36^ Therefore, it called for regulations to be regulated in the same way as conventional tobacco.^36^ This scientific information relating to the potential risks of HTPs must be informed to the public to eliminate misunderstandings about the safety of HTPs suggested by the advertisements of tobacco companies. In addition, regulations related to HTPs should be carefully considered in Japan. Recently, the Japanese government decided to strengthen the law to prevent passive smoking in public facilities used by many people from July 2019 to April 2020.^37^ However, this revision of the law has a critical flaw. The law regulates that eating and drinking are not allowed in rooms where smoking traditional combustible cigarettes are accepted. However, in the rooms intended for eating and drinking, smoking of HTPs is permitted due to the unclear risk of HTPs at the time the revision was discussed. Therefore, the smoking of HTPs in a closed environment in a restaurant is permitted by law. As mentioned above, there are health deterioration risks associated with the passive smoking of HTPs. Therefore, the law should be revised again to prohibit HTPs use in rooms of public facilities.

This study has several strengths but also limitations. As a strength, this study is the first to suggest the possibility that HTPs disrupt the decline in the smoking rate in Japan. In addition, as far as we know, this study is the first to clearly show the association between the use of HTPs, without the use of e-cigarettes, and SES because this study was conducted after relatively widespread use of HTPs in the market. Another strength is that this study used an adequate questionnaire to distinguish the use of HTPs and e-cigarettes: the questionnaire asked the respondents about their smoking status for each tobacco product, including the names of the products (ie, IQOS, Ploom Tech, and glo). It is considered difficult to distinguish between HTPs and e-cigarettes for people in general, and some e-cigarettes do not include any nicotine. However, the questionnaire was adequate for this study. There are some limitations to this study. First, there is the possibility of misclassification due to the use of a self-reported questionnaire. However, it is reported that tobacco use measured using self-reports tends to underestimate than actual smoking status.^38^ Therefore, our study may underestimate the prevalence of HTP users. In addition, we could not confirm the validity of the equivalent income variable. Although there was also a possibility of misclassification, the analyzed result was consistent with the results of other SES indices. Second, because this study used an online survey, there is the possibility of selection bias. According to the 2016 National Livelihood Survey, the distribution of household income was as follows: 6.2% for <1 million, 13.4% for 1.00–1.99 million, 13.7% for 2.00–2.99 million, 13.2% for 3.00–3.99, 10.4% for 4.00–4.99 million, 8.8% for 5.00–5.99 million, 7.7% for 6.00–6.99 million, 6.3% for 7.00–7.99 million, 4.9% for 8.00–8.99 million, 3.7% for 9.00–9.99 million, and 11.7% for ≥10 million.^39^ In our online survey, the distribution of income of the 5,000 surveyed population for corresponding income category was as follows: 7.3%, 6.0%, 12.6%, 14.5%, 12.7%, 11.2%, 8.2%, 7.0%, 4.7%, 4.4%, and 11.4%, respectively. These relatively similar distributions suggest that present results have a certain amount of external validity, although the generalizability of this study should be carefully considered.

### Conclusion

This study determined social differences in HTPs use. Younger people and more affluent people tended to use HTPs. Although smoking rates among these populations are generally lower, HTPs seem to disrupt the reduction in the smoking rate in Japan.

## APPENDIX A. SUPPLEMENTARY DATA

eTable 1. The distribution of educational attainment and occupation according to smoking status and the type of tobacco products among participants

eTable 2. Odds ratios of educational attainment and occupation for smokers (only combustible cigarette or HTP) versus non-smoker using multinomial logistic regression analysis

eTable 3. Odds ratios of educational attainment and occupation for HTP smokers versus only combustible cigarette smokers using logistic regression analysis
