# Supplementary material for: Heated Tobacco Products Have Reached Younger or More Affluent People in Japan
Source: J Epidemiol. 2021 Mar 5;31(3):187–93. doi: 10.2188/jea.JE20190260 (PMC7878708; doi:10.2188/jea.JE20190260)
Supplement: Supplementary file 1 [file je-31-187-s001.pdf]

eTable 1. The distribution of educational attainment and occupation according to smoking status and the type of tobacco products among participants

		Total (n=4,926)		Non-smokers (n=4,077)		Only combustible cigarette smokers (n=700)		HTP smokers[a] (n=149)	
		n	%	n	%	n	%	n	%
Men	Total	2,443	100	1858	76.1	472	19.3	113	4.5
	Education attainment								
	Junior high school and High school	660	27.0	494	26.6	142	30.1	24	21.2
	Technical college, 2-year college and Higher professional school	324	13.3	231	12.4	72	15.2	21	18.6
	University and Graduate school	1,456	59.6	1,131	60.9	257	54.5	68	60.2
	Other	3	0.1	2	0.1	1	0.2	0	0.0
	Occupation								
	Management, Professional, and Technical	682	27.9	506	27.2	138	29.2	38	33.6
	Clerical	328	13.4	247	13.3	60	12.7	21	18.6
	Sales and Service	339	13.9	242	13.0	74	15.7	23	20.3
	Production process, Transport, Cleaning, and Packaging, etc	133	5.4	95	5.1	35	7.4	3	2.7
	Transportation, Machine operation, Construction, and Mining	80	3.3	52	2.8	19	4.0	9	8.0
	Part-timer	143	5.8	116	6.3	25	5.3	2	1.8
	Inoccupation	402	16.5	331	17.8	66	14.0	5	4.4
	Other	336	13.8	269	14.5	55	11.7	12	10.6
Women	Total	2,483	100	2,219	89.4	228	9.2	36	1.5
	Education attainment								
	Junior high school and High school	788	31.7	678	30.5	102	44.7	8	22.2
	Technical college, 2-year college, and Higher professional school	835	33.6	746	33.6	79	34.7	10	27.8
	University and Graduate school	858	34.6	794	35.8	46	20.2	18	50.0
	Other	2	0.1	1	0.1	1	0.4	0	0.0

Occupation								
Management, Professional, and Technical	193	8.0	174	8.0	13	5.7	6	16.7
Clerical	445	17.9	396	17.8	39	17.1	10	27.8
Sales and Service	194	7.8	160	7.2	31	13.6	3	8.3
Production process, Transport, Cleaning, and Packaging, etc	43	1.7	34	1.5	8	3.5	1	2.8
Transportation, Machine operation, Construction, and Mining	10	0.4	9	0.4	1	0.5	0	0.0
Part-timer	284	11.4	259	11.6	24	10.5	1	2.8
Inoccupation	218	8.7	197	8.9	18	7.9	3	8.3
Other	1096	44.1	990	44.6	94	41.2	12	33.3

HTP, heated tobacco product.

[a]HTP smokers includes smokers using only HTPs or HTPs and other tobacco products.

eTable 2. Odds ratios of educational attainment and occupation for smokers (only combustible cigarette or HTP) versus non-smoker using multinomial logistic regression analysis

		OR for being only combustible cigarette smokers<br>aOR[a] (95% CI)	OR for being HTP smokers[b]<br>aOR[a] (95% CI)
Men	Education attainment		
(n=2,443)	Junior high school and High school	1.00 (reference)	1.00 (reference)
	Technical college, 2-year college and Higher professional school	1.09 (0.79–1.51)	1.76 (0.96–3.25)
	University and Graduate school	0.80 (0.63–1.01)	1.21 (0.75–1.96)
	Other	1.79 (0.15–20.7)	–
	Occupation		
	Management, Professional, and Technical	1.04 (0.73–1.46)	1.06 (0.60–1.86)
	Clerical	1.00 (reference)	1.00 (reference)
	Sales and Service	1.22 (0.83–1.79)	1.23 (0.66–2.29)
	Production process, Transport, Cleaning, and Packaging, etc	1.47 (0.91–2.39)	0.38 (0.11–1.32)
	Transportation, Machine operation, Construction, and Mining	1.44 (0.79–2.62)	2.20 (0.94–5.15)
	Part-timer	0.98 (0.58–1.65)	0.18 (0.04–0.77)
	Inoccupation	0.75 (0.49–1.12)	0.27 (0.10–0.74)
	Other	0.93 (0.62–1.40)	0.45 (0.21–0.95)
Women	Education attainment		
(n=2,483)	Junior high school and High school	1.00 (reference)	1.00 (reference)
	Technical college, 2-year college and Higher professional school	0.67 (0.49–0.92)	1.12 (0.43–2.87)
	University and Graduate school	0.42 (0.29–0.60)	1.49 (0.63–3.50)
	Other	6.49 (0.39–106.75)	–
	Occupation		
	Management, Professional, and Technical	0.79 (0.41–1.53)	1.24 (0.44–3.52)
	Clerical	1.00 (reference)	1.00 (reference)
	Sales and Service	2.11 (1.26–3.52)	0.70 (0.19–2.59)

Production process, Transport, Cleaning, and Packaging, etc	2.51 (1.08–5.88)	1.06 (0.13–8.68)
Transportation, Machine operation, Construction, and Mining	1.22 (0.15–10.18)	–
Part-timer	0.94 (0.55–1.61)	0.15 (0.02–1.19)
Inoccupation	1.00 (0.55–1.81)	0.63 (0.17–2.37)
Other	0.93 (0.62–1.39)	0.59 (0.25–1.41)

aOR, adjusted odds ratio; CI, confidence interval; HTP, heated tobacco product.

[a] Adjusted for age.

[b]HTP smokers includes smokers using only HTPs or HTPs and other tobacco products.

eTable 3. Odds ratios of educational attainment and occupation for HTP smokers versus only combustible cigarette smokers using logistic regression analysis

		OR for being HTP smokers[b]
		aOR[a] (95% CI)
Men	Education attainment	
(n=585)	Junior high school and High school	1.00 (reference)
	Technical college, 2-year college and Higher professional school	1.80 (0.90–3.59)
	University and Graduate school	1.49 (0.87–2.55)
	Other	–
	Occupation	
	Management, Professional, and Technical	1.14 (0.59–2.22)
	Clerical	1.00 (reference)
	Sales and Service	1.09 (0.52–2.25)
	Production process, Transport, Cleaning, and Packaging, etc	0.30 (0.08–1.12)
	Transportation, Machine operation, Construction, and Mining	2.21 (0.89–6.11)
	Part-timer	0.27 (0.06–1.33)
	Inoccupation	0.58 (0.19–1.78)
	Other	0.48 (0.20–1.14)
Women	Education attainment	
(n=264)	Junior high school and High school	1.00 (reference)
	Technical college, 2-year college and Higher professional school	2.11 (0.75–5.93)
	University and Graduate school	4.12 (1.55–11.00)
	Other	–
	Occupation	
	Management, Professional, and Technical	3.21 (0.75–13.70)
	Clerical	1.00 (reference)
	Sales and Service	0.40 (0.09–1.76)

Production process, Transport, Cleaning, and Packaging, etc	0.56 (0.06–5.63)
Transportation, Machine operation, Construction, and Mining	–
Part-timer	0.23 (0.03–2.05)
Inoccupation	0.75 (0.16–3.61)
Other	0.62 (0.23–1.70)

aOR, adjusted odds ratio; CI, confidence interval; HTP, heated tobacco product.

[a] Adjusted for age.

[b]HTP smokers includes smokers using only HTPs or HTPs and other tobacco products.
